# Trees, Climate Change, and Health: An Urban Planning, Greening and Implementation Perspective

**DOI:** 10.3390/ijerph20186798

**Published:** 2023-09-21

**Authors:** Alistair Woodward, Andrea Hinwood, Daniel Bennett, Brenton Grear, Sotiris Vardoulakis, Neha Lalchandani, Katrina Lyne, Carmel Williams

**Affiliations:** 1 School of Population Health, University of Auckland, Auckland 1010, New Zealand; 2 United Nations Environment Programme, Nairobi 00100, Kenya; 3 Australian Institute of Landscape Architects, Adelaide 5001, Australia; 4 Green Adelaide, Department for Environment and Water, Adelaide 5000, Australia; 5 Healthy Environments and Lives (HEAL) Network, Australian National University, Canberra 0200, Australia; 6 School of Public Health, The University of Adelaide, Adelaide 5005, Australia; 7 Centre for Health in All Policies Research Translation, Health Translation SA, SAHMRI, Adelaide 5000, Australia

**Keywords:** public health, environmental health, urban environment, green space, environmental policy, sustainable cities

## Abstract

The In Conversation: Boundary, Spanners, Thinkers and Policy Actors Round Table Series provides a platform for researchers, policy actors, and implementation experts to elevate discussion on emerging issues, present new and upcoming research, and facilitate conversations around impacts and possible solutions. This brief report, on trees, climate change, and health, reflects a conversation between the authors of this paper, along with supporting literature. It explores the potential of green spaces and trees as a viable strategy to address climate change challenges and simultaneously improve population health, well-being, and health equity. In particular, it highlights the public health benefits of trees and green space, the challenges faced in urban areas, and opportunities for the protection, maintenance and regeneration of urban green space.

## 1. Introduction

The term green infrastructure, also known as green space, refers to networks of natural, designed, or cultivated vegetation spaces in public and private areas in urban environments [1], including parks, gardens and streetscapes. Green spaces provide important environmental, ecosystem, economical, and social services [2,3,4,5]. Trees are a notable component of urban green spaces, and are often viewed as valuable natural resources that enhance the liveability of cities [6,7,8]. Trees and green spaces also have important benefits for human health in urban environments. Research has demonstrated the positive impact of trees and green spaces on physical and mental health, as well as overall social wellbeing [9,10,11], making them a potentially effective public health intervention. Enhanced efforts to optimise the design of green spaces and promote the establishment and protection of trees and other vegetation are necessary to realise their health and environmental benefits in urban settings. Additionally, initiatives to promote the establishment and protection of trees and other vegetation within cities are crucial. Such efforts will also support the realisation of the United Nations’ (UN) Sustainable Development Goals [12], which encompass a wide range of global objectives aimed at creating more sustainable and equitable urban environments. 

## 2. The Power of Trees

The World Health Organization (WHO) recognises the importance of green spaces and natural environments for human health [13]. Trees and green spaces provide numerous environmental benefits by enhancing air quality, mitigating the urban heat island effect, reducing energy consumption, sequestering carbon, and aiding water management, making them a crucial component of climate change mitigation and adaptation strategies in urban areas [2,3,4,14,15]. Research has linked exposure to trees and green spaces with improved mental health, reduced stress levels, reduced loneliness, lower all-cause mortality, and lower rates of obesity and chronic diseases [16,17,18,19]. 

Trees add character and identity to places, with green spaces providing an opportunity for recreation and social connections in cities, and yielding cultural, behavioural, and community benefits [5]. Green settings serve as focal points for communities, bringing people together, fostering a sense of belonging and unity, which further contributes to the cultural fabric of cities. Additionally, when individuals have easy access to green spaces, this phenomenon encourages residents to engage in physical activity (including walking and cycling) and increased time spent outside in nature [11]. This offers an escape from the hustle and bustle of urban city living and promotes healthier habits, ultimately contributing to the wellbeing and vitality of city residents. 

A range of select examples demonstrate the benefits of trees and green space for human health:

(1) The Robert Taylor Homes project in Chicago serves as a compelling example of how the presence of green spaces can have a significant impact on the behaviour and safety of residents. Those living in apartments overlooking green space were 20 to 30% less likely to exhibit aggressive and violent behaviour than those who did not [20]. More recent research underscores the positive effects of green space on reducing aggressive and violent tendencies in urban communities [21].
(2) The emerald ash borer outbreak in the United States represents a significant ecological and public health challenge. The invasive insect species caused deaths of millions of ash trees across various regions of the country. Tree loss in the affected counties was associated with more than 20,000 excess deaths due to cardiovascular and lower respiratory tract illness from 1990 to 2007, showing that loss of trees is likely harmful to human health in ways we do not yet fully understand and is associated with increased mortality [22,23].
(3) Acute effects of green space on human physiology, specifically in urban environments, have been demonstrated. These include reductions in stress responses (for example, heart rate slowing, blood pressure reductions, and altered patterns of cortisol release) after exposure to green space in urban areas [24,25,26]. Living in an area with increased tree canopy cover has also been associated with reduced incidence and prevalence of cardiometabolic conditions, such as diabetes, hypertension and cardiovascular diseases, in New South Wales, Australia [27].

In essence, trees and other green space can offer enormous benefits for the environment and the health of city dwellers. 

## 3. What Is the Problem?

The triple planetary crisis of climate change, biodiversity loss, and pollution is threatening the health of Earth and its inhabitants [28,29]. This crisis refers to long-term alterations in global temperature and weather patterns; biodiversity loss, which involves the decline and extinction of various species and ecosystems; and pollution, which includes the contamination of air, water, and soil by harmful substances. The deterioration of the natural environment is undermining progress towards the UN Sustainable Development Goals related to poverty, hunger, health, water, cities, climate, oceans, and land [12]. For example, ecosystem degradation and biodiversity loss exacerbate the risk of zoonotic disease emergence and spread, while climate instability threatens food security [29]. Global action is necessary to reverse these trends and maintain the health of the planet. 

The majority of the world’s population lives in cities [30]. Although the benefits of urban greening are well-documented [31], lack of greening is also affected by insufficient water, especially in low-income or poor countries. Reasons for this are numerous; for example, rapid urban growth and densification, the competing use of urban areas, particularly for vehicular transport systems, and unsympathetic urban planning and policy settings [32]. The lack of protection of existing trees is a common problem [33,34], indicating that established greenery is often at risk of removal due to urban development or neglect. Inappropriate greening is associated with pollen and biogenic emissions of volatile organic compounds [4], while inadequate greening compounds the urban heat island effect, as demonstrated in Canada [35], Australia [36], and China [37]. Higher temperatures caused by the urban heat island effect have significant consequences for human health and wellbeing, including heat-related illnesses and decreased comfort [38,39], as well as negative social and economic effects [40,41]. The urgency of addressing the planetary crises through urban greening supported by appropriate environmental policies is evident. 

## 4. The Way Forward 

Planting the right trees in the right places, and looking after them properly, can be a powerful strategy to promote public health and wellbeing. Urban greening through planting and maintaining trees has multifaceted co-benefits, particularly with respect to climate change by sequestering carbon dioxide through photosynthesis. This helps reduce greenhouse gas emissions in urban environments, contributing to global efforts to combat climate change. Importantly, trees can produce increasing benefits as they grow and mature, making them one of the few public assets that appreciate in value over time [5]. For example, the air quality improvement, shade provision, and aesthetic appeal of mature trees through investing in planting and caring for them can be a long-term strategy that pays dividends over time. 

As mentioned above, a range of factors contribute to inadequate coverage of trees and green space in many urban areas. Effective responses to these challenges require collaborative approaches in the form of multi-sectoral reforms, such as the “environment in all policies” approach [42]. This approach seeks to make environmental considerations explicit during policy and decision making across sectors and requires multi-disciplinary collaboration and coordination. Synergistic approaches involving urban planners, governments, developers, and the community will help to identify, emphasise, and realise the co-benefits of urban greening for human health, society, and the environment, thus helping achieve several targets of the UN Sustainable Development Goals [12]. Also, connection to Country and engagement of First Nations communities and First Nations’ ways of thinking, being, and knowing should be at the centre of city planning, since systems thinking and long-term intergenerational perspectives, which feature so strongly in indigenous worldviews, underpin a sustainable and comprehensive approach to human and environmental health [43,44,45]. 

Green space research holds significant promise in addressing the complex environmental and social challenges facing urban areas worldwide. It not only offers a strategic approach to combatting prevalent environmental hazards but also carries cultural, behavioural, and community significance. As a result, many steps have been taken to promote urban trees and green space more broadly, particularly as urban populations grow and cities densify. Green infrastructure is specifically listed as Target 11.7 in the UN Sustainable Development Goals: “By 2030, providing universal access to safe, inclusive and accessible, green and public spaces, in particular for women and children, older persons and persons with disabilities” [46]. The United Nations has designated 2021–2030 as the “decade for ecosystem restoration”, with numerous initiatives to improve local ecosystems, including in urban areas, underway worldwide [47]. The World Health Organization’s Healthy Cities initiative also calls for healthy urban planning and design, and investment in green policies to promote health and wellbeing in urban environments [48]. These global policy actions can translate into the strategic use of green spaces and tree plantations as a pivotal asset in advancing and facilitating environmental rejuvenation and fostering public health and well-being.

Likewise, a range of initiatives have been designed to support the expansion and enhancement of green space at local and national levels. For example, National Park Cities is an international grassroots movement intended to make cities greener, healthier, and wilder across public and private spaces [49]. Tree planting efforts are a key focus for many countries, such as Pakistan’s goal to plant ten billion trees by 2023 [50]. Water- and biodiversity-sensitive urban design is promoted by initiatives such as Green Adelaide, an urban Landscape Board which was established to foster cooler, greener, wilder, and climate-resilient metropolitan areas in South Australia [51]. Despite these schemes, sustained cross-sectoral effort is needed to ensure the necessary increases in numbers of trees and quality green space in urban areas, for example, planning reforms; the strengthening of tree protection regulations; increased realisation of the human health, environmental, and social value of trees; and continued investment in quality green space on both public and private land. 

Regardless of the setting, it is important that initiatives are actioned according to the greatest need; a “business as usual” approach is not sufficient, and evidence-informed strategies are essential. Importantly, the hottest, least green spaces should be prioritised, as the greening of these areas will have the greatest impact. This process requires the heat mapping of urban areas and equitable planning and response processes. Disparities between wealthier suburbs and poorer suburbs regarding tree cover are well-documented, and should be tackled in the design of greening programs to reduce health inequity [52,53]. In addition, it is important that progress is mapped and tracked over time, particularly so that successful greening approaches can be learned from, scaled up, and replicated in other settings. Local governments and urban planners have a critical role to play in greening strategies, with good land use planning and appropriate regulation essential to protect, maintain, and restore greening in urban environments. 

While urban greening is a powerful strategy through which to support environmental, planetary, and human health, unintended consequences must be considered. For example, falling limbs from poorly maintained trees can lead to injury, and inappropriate vegetation species selection may risk exacerbations of allergic disease and compromise local air quality [54]. Limiting the range of urban vegetation species undermines biodiversity and risks the loss of canopy cover should plant disease or climate-related threats occur. Greening and land use policies that ignore the potential hazards or unintended consequences posed by vegetation species selection can therefore be detrimental to public health and wellbeing, calling for a comprehensive and considered approach. 

Communities are also well-placed to support greening efforts. Increasing public awareness of the health, social, environmental, and economic benefits of trees and green spaces can be achieved through public education campaigns and community outreach programs. Changing community attitudes and expectations can be a powerful driver for improved access to quality green space. The engagement of the community is essential given that a large proportion of urban green space is found on private property, with protection and enhancement of these areas being just as important as that of public land. Additionally, it is important to recognise the value of international collaborations and knowledge sharing to encourage sustainable urban development and green practices to address global challenges like climate change. Urban planners and policy makers around the world can learn from each other’s successes and challenges in urban planning strategies and greening efforts on a global scale for creating sustainable, healthy, and resilient cities. However, we are cognizant that the discussion between researchers, policy actors, and implementation experts and the ideas presented in this report are limited to the Australian context. It is imperative that this dialogue extends to the international stage, transcending national borders and recognising that climate change is a pressing global issue. 

## 5. Conclusions

This brief report has synthesised a conversation between the authors regarding the value of trees and green space. It highlights the critical role of urban planning and public health in protecting, valuing, and regenerating trees and green space, to support connections with nature and realise co-benefits for human, environmental, and planetary health. An awareness of forests, trees, and green space is fundamental for the conservation of land and protection of clean air and good health. A range of challenges exist, including the need for increased housing and densification of cities, inadequate tree protection regulations, and failure to appreciate the multi-faceted value of trees and green space, as well as the consequences of poor tree canopy design and vegetation species selection. We recommend planning reforms, improved regulations, changing community attitudes through education and public health campaigns, and investment in quality green spaces to strengthen urban greening and make cities healthier and more sustainable for all residents. Increased awareness among governments, urban planners, developers, and the community, along with responsive policy development and implementation, are necessary to support this endeavour. 

Another critical area is the role of community members advocating for the protection, maintenance, and regeneration of urban green space. If people love and value the places they live in, they will lobby for their regeneration and support efforts to care for and maintain green spaces. Connection with country and nature is an integral component of many First Nations cultures and knowledge systems, which can provide inspiration and knowledge contributing to a more holistic approach to health and wellbeing. Overall, success requires continued collaboration between governments, urban planners, environmental and public health practitioners, the private sector, community organisations, and urban dwellers to realise the potential of nature-based solutions and the numerous co-benefits of urban trees and green space. 

## Data Availability

Not applicable.
